# House dust mite immunotherapy: A real‐world, prescription data‐based analysis

**DOI:** 10.1002/clt2.12382

**Published:** 2024-07-11

**Authors:** R. Mösges, H. Richter, A. Sager, J. Weber, T. Müller

**Affiliations:** ^1^ ClinCompetence Cologne GmbH Cologne Germany; ^2^ Institute of Medical Statistics and Computational Biology (IMSB) University of Cologne Cologne Germany; ^3^ RWS Epidemiology IQVIA Frankfurt Germany; ^4^ Medical Department LETI Pharma GmbH Ismaning Germany

**Keywords:** allergen immunotherapy, allergic asthma, allergic rhinitis, house dust mite, real‐world analysis

## Abstract

**Background:**

House dust mite (HDM) sensitisation can contribute to the development of allergic rhinoconjunctivitis (AR) or allergic asthma (AA). As treatment, allergen immunotherapy (AIT) is a promising approach, since it aims building immunotolerance against allergens, therewith establishing long‐term efficacy. The evaluation of AIT has been investigated in many randomised controlled trials, whereas few real‐world evidence studies are available.

**Methods:**

We used data from the longitudinal prescription data base IQVIA™ LRx. Data on initial AIT prescriptions against HDM from January 2009 to December 2013 was analysed regarding treatment (subcutaneous AIT with either depigmented polymerised allergen extract [dSCIT] or other allergens [oSCIT], or sublingual immunotherapy [SLIT]) and treatment duration. Treatment groups were compared with a control group of AR patients not receiving AIT. Data on symptomatic medication was collected until February 2017 and progression of AR and AA was compared.

**Results:**

Data of 7260 patients with AIT prescriptions and of 21,780 control patients was analysed. AIT was associated with a significant decrease of AR medication intake compared with control (dSCIT: −34.0%, *p* < 0.0001; oSCIT: −25.7%, *p* < 0.0001; SLIT: −37.7%, *p* = 0.0026). In asthmatics, SCIT was associated with a significant decrease of asthma medication compared with control (dSCIT: −45.2%, *p* < 0.0001; oSCIT: −32.9%, *p* < 0.0001). Further, a significantly reduced likelihood for onset of asthma medication was demonstrated in patients treated with SCIT compared with controls (dSCIT OR: 0.759, *p* = 0.0476; oSCIT OR: 0.815, *p* = 0.0339).

**Conclusion:**

Real‐world data analyses indicate that AIT, particularly given via a subcutaneous route, reduces the need of medication against AR and AA and might delay the onset of asthma medication in patients with AR.

## 1 INTRODUCTION

Allergen immunotherapy (AIT) is the only causal and preventive allergic disease modifying therapy available.^1^, ^2^, ^3^ By repeatedly exposing the patient to the allergen, the immune system becomes desensitised to it. For AIT, several standardised products are available, differentiated by the route of application as subcutaneous (SCIT) or sublingual immunotherapy (SLIT). For SCIT, unmodified allergens or chemically modified allergens (allergoids) are used.^2^ SLIT, on the other hand, is almost always available in the form of unmodified allergens. SCIT is administered by the physician and requires regular visits during the maintenance phase, whereas SLIT is taken by the patient at home without further supervision by the prescribing physician.

Results from many randomised controlled trials (RCTs) investigating the efficacy and safety of AIT have been published within the last decades. However, controlled studies feature inherent results bias since the patients included are selected according to clearly defined inclusion and exclusion criteria and are instructed and monitored during the study.^4^, ^5^, ^6^ Therefore, the results of RCTs are only representative for about 5% of the general population, which differs in characteristics such as body mass index, comorbidities, or age.^7^ Consideration of the heterogeneity of the population and evidence of reliable real‐world evidence (RWE) studies are therefore essential to assess the efficacy and safety of AIT under real world conditions. RWE aims to support RCT evidence by analysing data from daily clinical practice and has been demonstrated to be very helpful, reliable and complementary to controlled trials in the field of AIT, especially allowing insights into effectiveness in children and adolescents.^8^, ^9^, ^10^

House dust mites (HDMs) have a well‐established causal role in patients with persistent allergic respiratory diseases, such as allergic rhinoconjunctivitis (AR) and allergic asthma (AA).^11^ The efficacy of AIT in patients with allergic respiratory conditions has long been acknowledged using national and international guidelines.^1^, ^2^ Several double‐blind, placebo controlled (DBPC) randomised clinical trials of SCIT and SLIT with HDM allergen extracts have shown that this treatment is effective and safe.^12^, ^13^, ^14^, ^15^, ^16^, ^17^, ^18^, ^19^, ^20^, ^21^, ^22^, ^23^ On the other hand, RWE studies assessing the efficacy of AIT treatment in daily life are rare.^24^

Therefore, the aim of this RWE study was to analyse large data on longitudinal prescriptions of depigmented polymerised HDM allergen extracts (dSCIT), selected other HDM‐SCIT (oSCIT) or selected HDM‐SLIT products (SLIT) to assess the therapeutic effects on AR progression and asthma progression or onset.

## 2 METHODS

The current analysis was based on the prescription data base IQVIA™ LRx, which collects data of statutorily insured patients in Germany. The selected data set covers 60% of the German statutory population, incorporating demographic (age, sex) and prescription related information (product, substance, form, package size, etc.) from 2008 onwards. There are no data available for diagnoses and laboratory tests.

**FIGURE 1:**
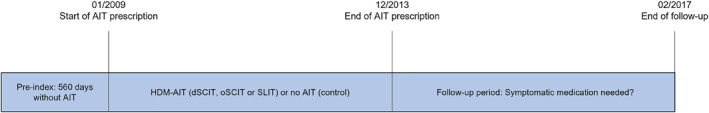
Timeline of the study including pre‐index, treatment period, and follow‐up period.

Prescriptions of AIT (EphMRA ATC: V1A) in the database were identified for HDM. The selection timespan from January 2009 to December 2013 was used and aligned with the start of the ‘main allergy season’ of HDM from September–December. Product and prescription date were designated as the index product and the index date, respectively (Figure 1). Development of AR was assessed by prescriptions of symptomatic medication (nasal corticosteroids (NCS), oral/nasal antihistamines). For identification and outcome assessment of AA, prescription of inhaled corticosteroids (ICS), ICS/long acting ß‐agonists fixed combination and anti‐IgE/IL‐5 biologicals were used. Due to several reasons, the index period did not exceed December 2013. First, the database could not be analysed beyond February 2017 on account of the release of NCS from prescription binding in March 2017. Second, there would otherwise not have been a sufficient observation time post index to meet the requirements of both treatment and follow‐up periods (at least 4 years in total).

Patients were selected if they met the following criteria:

- Initial prescription of AIT from January 2009 to December 2013
- No AIT in the previous 560 days and no HDM‐AIT at any time before inclusion in the study
- Two or more seasonal cycles of AIT between January 2009 and December 2013 (one seasonal cycle for HDM is equivalent to 1 year, from January to December)
- Patient aged ≥5 years at index date
- Patient can be followed up for at least 2 years after the end of AIT (follow‐up period)

Based on differences regarding chemical modifications and administration routes, the following three product groups were defined and analysed:

- Subcutaneous immunotherapy with depigmented polymerised allergen extract (dSCIT)
  - DEPIGOID^®^ from LETI Pharma
- Other subcutaneous immunotherapy (oSCIT), including native extracts and non‐depigmented allergoids
  - ALK Depot^®^ from ALK‐Abelló, Acaroid^®^ from Allergopharma, and Purethal^®^ from HAL Allergy
- Sublingual immunotherapy (SLIT)
  - Oralvac^®^ from Bencard Allergy, Sublivac^®^ from HAL Allergy, and Acarizax^®^ from ALK‐Abelló (the latter not being analysed during this study due to its late marketing authorisation date in 2015)

For each AIT prescription, the application duration was estimated using the manufacturer's recommendation. For subcutaneous treatments, the duration was based on the total package volume and volume per application considering differential application volumes during up‐dosing. Patients were observed until the first of the following occurred:

- Complete cessation of the AIT
- Grace period (end to next start of successive prescriptions) exceeding 90 days
- End of patient observability

The treatment groups were compared to a control group with non‐AIT patients who must have had at least two prescriptions of symptomatic AR medication 560 days apart.

Progression of AR and AA was analysed by comparing the number of prescriptions for symptomatic medication in the follow‐up period between the AIT‐treatment group and the control group. The onset of AA was measured by first prescriptions of asthma medication in the follow‐up period. For statistical analysis, logistic regression (binary outcome variables) and Poisson regression (count variables) were used.

## 3 RESULTS

In total, 7260 patients were included in the analyses (2110 treated with dSCIT, 4928 treated with oSCIT and 222 treated with SLIT) and 21,780 patients without AIT were selected to act as a control group.

**FIGURE 2:**
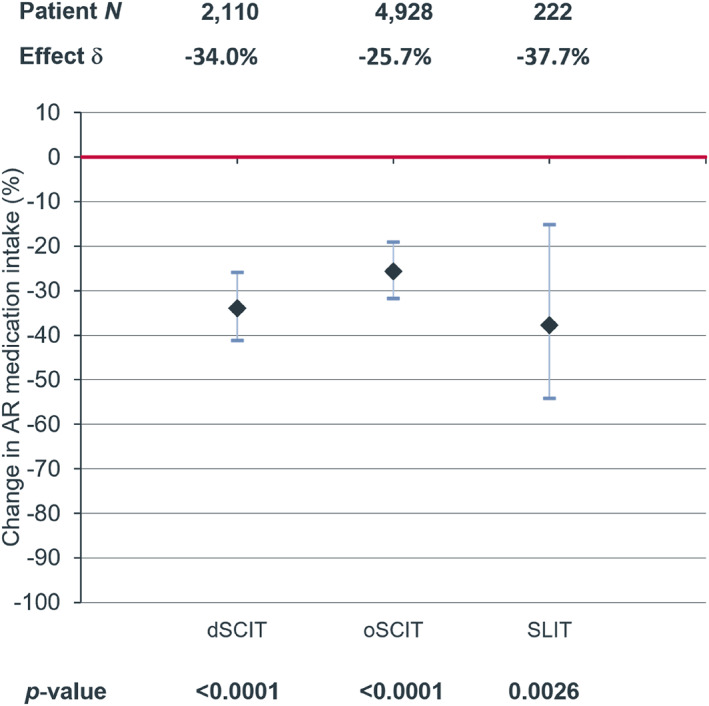
Decrease (%) of prescriptions of AR medication in the follow‐up period after HDM‐AIT treatment with dSCIT, oSCIT and SLIT compared to control without AIT treatment (red line). AIT, allergen immunotherapy; AR, allergic rhinoconjunctivitis; HDM, house dust mite; SLIT, sublingual immunotherapy.

**FIGURE 3:**
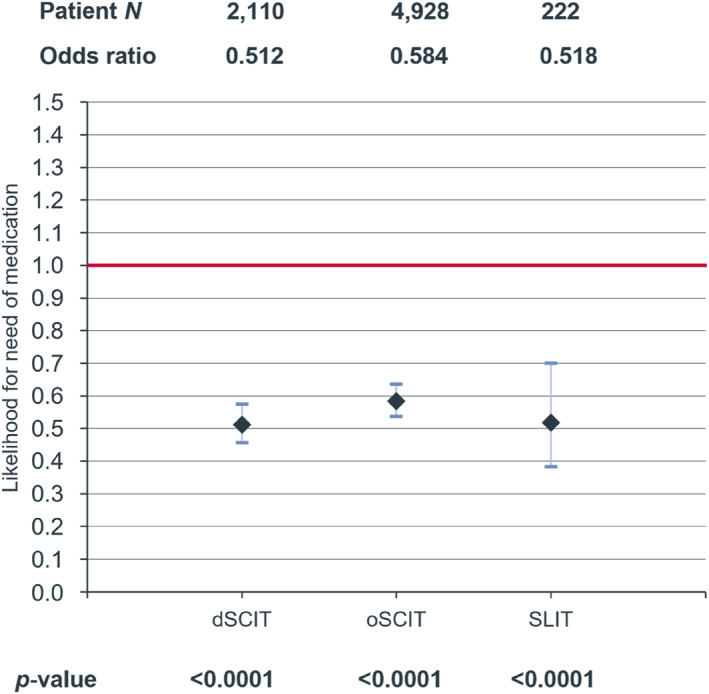
Likelihood (with 95% CI) for the need of AR medication in the follow‐up period after HDM‐AIT treatment with dSCIT, oSCIT and SLIT compared to control without AIT treatment (red line). AIT, allergen immunotherapy; AR, allergic rhinoconjunctivitis; HDM, house dust mite; SLIT, sublingual immunotherapy.

Overall, AIT treatment with HDM allergen extract was associated with a significant decrease in AR medication intake during the follow‐up period compared with the non‐AIT control (dSCIT: −34.0%, *p* < 0.0001; oSCIT: −25.7%, *p* < 0.0001; SLIT: −37.7%, *p* = 0.0026) (Figure 2). The treatment was also associated with a significantly reduced likelihood for the need of AR medication (dSCIT OR:0.512, *p* < 0.0001; oSCIT OR:0.584, *p* < 0.0001; SLIT OR:0.518, *p* < 0.001) (Figure 3).

**FIGURE 4:**
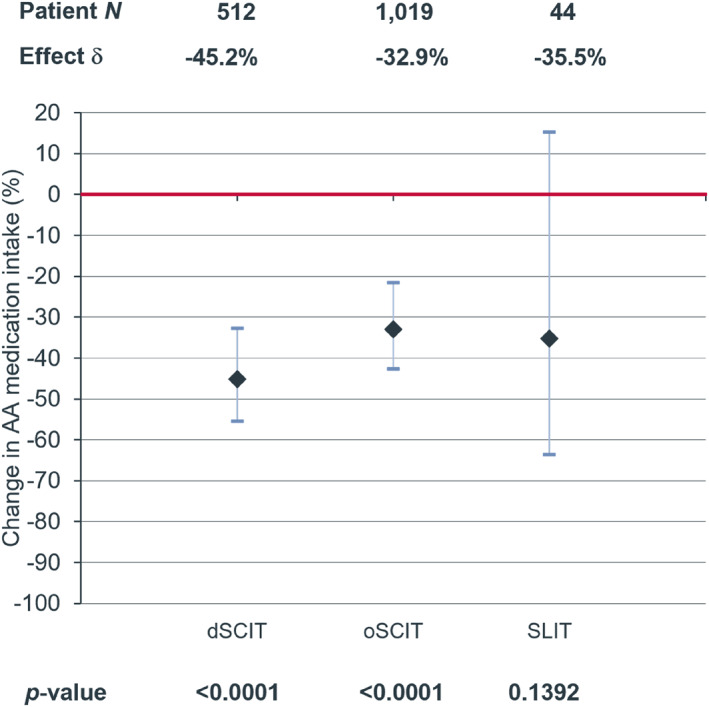
Decrease (%) of prescriptions of asthma medication in the follow‐up period in patients with asthma at baseline, after HDM‐AIT treatment with dSCIT, oSCIT and SLIT compared to control without AIT treatment (red line). AIT, allergen immunotherapy; HDM, house dust mite; SLIT, sublingual immunotherapy.

In patients with asthma at baseline (512 in the dSCIT group, 1019 in the oSCIT group and 44 in the SLIT group), AIT was also associated with a considerable decrease of asthma medication compared to the control group with 5543 asthma patients (dSCIT: −45.2%, *p* < 0.0001; oSCIT: −32.9%, *p* < 0.0001; SLIT: −35.5%, *p* = 0.1392) (Figure 4). This effect was significant for dSCIT and oSCIT but not for SLIT.

**FIGURE 5:**
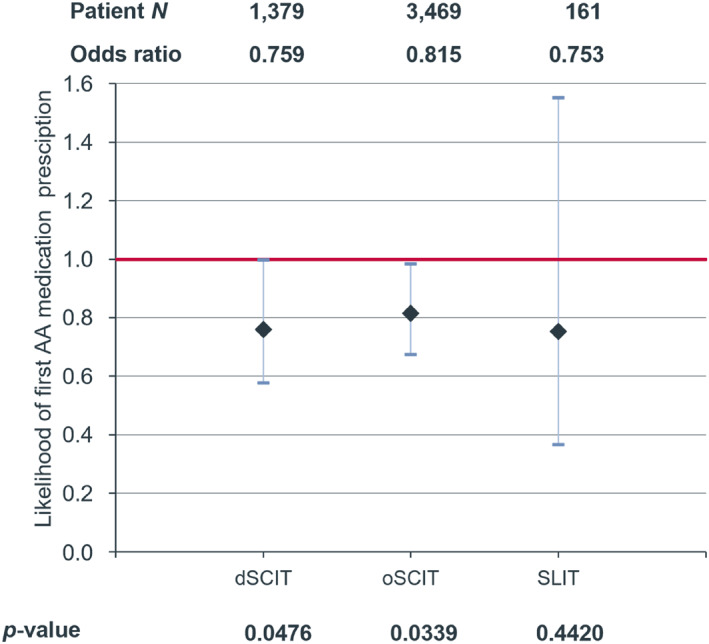
Likelihood (with 95% CI) for the first prescription of asthma medication in the follow‐up period after HDM‐AIT treatment with dSCIT, oSCIT and SLIT compared to control without AIT treatment (red line). AIT, allergen immunotherapy; HDM, house dust mite; SLIT, sublingual immunotherapy.

The likelihood for first prescription of asthma medication after AIT in patients without asthma at baseline (1379 in the dSCIT group, 3469 in the oSCIT group and 161 in the SLIT group) was significantly reduced in the dSCIT and oSCIT groups but not in the SLIT group (dSCIT OR: 0.759, *p* = 0.0476; oSCIT OR: 0.815, *p* = 0.0339; SLIT OR: 0.753, *p* = 0.4420) (Figure 5).

## 4 DISCUSSION

RWE studies are increasingly regarded to play an important role in the future and will complement RCTs in the assessment of safety and effectiveness in order to provide a comprehensive picture of the ‘real world’.^7^, ^25^, ^26^, ^27^ Paoletti et al.^7^ recently published a systematic review of observational studies of AIT calling for appraisal of the quality and importance of RWE in AIT. They underline the need for further collaborations to standardise the methodology for RWE studies. In this regard, even regulatory bodies such as the Food and Drug Administration are convinced of the added value of RWE studies and are working on the framework of the ‘Real World Evidence Programme’.^28^, ^29^

Based on the IQVIA^TM^ LRx prescription database, we analysed in the present study the effect of treatment with different forms of HDM‐AIT compared to a control group not receiving AIT on AR and AA. All forms of AIT were associated with a significant decrease of AR medication intake during the follow‐up period, which indicates strong efficacy of the AIT treatment. The same positive effect was seen for asthma medication in patients suffering from AA already before the start of the AIT. Furthermore, in patients without AA at baseline, the risk for the first asthma medication during the follow‐up period was significantly reduced with AIT compared to allergic patients who were not treated with AIT.

The study includes some limitations. As IQVIA™ LRx is a prescription database, it does not contain diagnostic information and all estimations must be based on prescriptions reimbursed by statutory health insurances only. Furthermore, during the end of the observational period, some AR medications were out of prescription and only available as Over‐the‐Counter (OTC) products. However, as the Rx‐to‐OTC switch affected all observed groups of the current analysis, effects on decline of prescriptions of AR medication will be comparable between groups and should not induce a bias on results. Conclusions regarding the effects of SLIT on asthma were limited due to the small size of this treatment group (*n* = 44), which did not allow a profound statistical analysis. The reason for the low number of SLIT treated patients was the fact that Acarizax^®^ was only introduced to the market after 2015, so that data of participants applying this product could not be included into the analyses. Thus, only data on the HDM‐products Oralvac^®^ and Sublivac^®^ were analysed during the observation timespan. Of note, as those two products are no longer available on the German market, interpretation of results of this subgroup should be made with caution.

Our RWE results clearly confirm the findings of other clinical studies.^21^, ^22^, ^23^ In two DBPC phase III studies in which all included patients were diagnosed with mild/moderate asthma and AR caused by HDM, the amount of allergen needed to obtain a positive bronchial provocation test was increased more than two‐fold compared to control after 1 year of AIT with depigmented polymerised HDM or *Dermatophagoides pteronyssinus* allergen extract.^21^, ^22^ Furthermore, in an open, prospective, controlled study with children suffering from HDM‐induced AA, a significant increase in allergen dose needed to induce a response in bronchial provocation concentration was seen already after 4 months of treatment with depigmented polymerised allergen extract from *D*. *pteronyssinus*. Similarly, the efficacy and safety of oSCIT products have been demonstrated in clinical studies.^15^, ^30^, ^31^, ^32^ An early Cochrane review^33^ that examined 54 RCTs of specific immunotherapy in asthma confirmed the efficacy of immunotherapy in asthma as evidence A. Similar effects were also found in an earlier RWE study.^24^ The current study has demonstrated that RWE may serve as a valid source for evaluating the clinical efficacy of AIT as the only existing disease modifier in allergic diseases.

## AUTHOR CONTRIBUTIONS

**R. Mösges**: Conceptualization; writing – original draft; formal analysis. **H. Richter**: Writing – original draft, formal analysis. **A. Sager**: Conceptualization; writing – original draft; formal analysis. **J. Weber**: Writing – review & editing. **T. Müeller**: Writing – review & editing.

## CONFLICT OF INTEREST STATEMENT

RM reports personal fees from ALK, grants from ASIT biotech, personal fees from allergopharma, personal fees from Allergy Therapeutics, grants and personal fees from Bencard, grants and personal fees from Inmunotek, grants from Leti, grants, personal fees and non‐financial support from Lofarma, non‐financial support from Roxall, grants and personal fees from Stallergenes, grants from Optima, personal fees from Friulchem, personal fees from Hexal, personal fees from Servier, personal fees from Klosterfrau, non‐financial support from Atmos, personal fees from Bayer, non‐financial support from Bionorica, personal fees from FAES, personal fees from GSK, personal fees from MSD, personal fees from Johnson&Johnson, personal fees from Meda, personal fees and non‐financial support from Novartis, non‐financial support from Otonomy, personal fees from Stada, personal fees from UCB, non‐financial support from Ferrero, grants from Hulka, personal fees from Nuvo, grants and personal fees from Ursapharm, personal fees from Menarini, personal fees from Mundipharma, personal fees from Pohl‐Boskamp, grants from Cassella‐med GmbH & Co. KG, personal fees from Laboratoire de la Mer, personal fees from Sidroga, grants and personal fees from HAL BV, personal fees from Lek, personal fees from PRO‐AdWise, personal fees from Angelini Pharma, grants and non‐financial support from JGL, grants and personal fees from bitop, grants from Sanofi, personal fees from Menarini, outside the submitted work. HR is employee of IQVIA, Frankfurt/Main, Germany. AS is an employee of LETI Pharma GmbH. JW is an employee of LETI Pharma GmbH. TM is an employee of LETI Pharma GmbH.

## Data Availability

Data sharing is not applicable to this article as no new data were created or analysed in this study.
